# Smoking, oxidative stress and inflammation: Impact on resting energy expenditure in diabetic nephropathy

**DOI:** 10.1186/1471-2369-6-13

**Published:** 2005-11-22

**Authors:** Rajiv Agarwal

**Affiliations:** 1 Department of Medicine, Indiana University and RLR VA Medical Center, Indianapolis, Indiana, USA

## Abstract

**Background:**

Inflammation is associated with increased resting energy expenditure (REE) in patients with chronic kidney disease. Oxidative stress, on the other hand, appears not to increase REE. Smoking is a common mechanism for generating oxidative stress and inflammation. Whether smokers have increased REE and if so, whether it is accounted for by the pro-oxidant and inflammatory state is not known.

**Methods:**

A case control study of 11 smokers and 24 non-smokers with overt diabetic nephropathy was performed to evaluate the chronic effect of smoking on REE. REE (indirect calorimetry), glomerular filtration rate (iothalamate clearance), markers of oxidative stress (urinary and plasma malondialdehyde (MDA), and protein carbonyls) and inflammation (C-reactive protein, tumor necrosis factor-alpha, interleukin-6) were measured on two occasions four months apart.

**Results:**

Biomarkers of inflammation (C-reactive protein) and oxidative stress (urinary and plasma MDA) were increased in smokers. REE was increased in smokers, 24.3 kcal/kg/day compared to 21 kcal/kg/day (p = 0.009) in non-smokers. After adjusting for age, GFR, MDA, C-reactive protein, and hemoglobin A_1_C the difference in REE between the two groups persisted (adjusted difference 3.51 kcal/kg/d, 95% confidence interval 0.59 – 6.45, p = 0.020).

**Conclusion:**

Patients with overt diabetic nephropathy who smoke have a higher REE, oxidative and inflammatory state. Elevated REE is not attributable to heightened oxidative stress and inflammatory state. Smoking is an independent risk factor for elevated REE in patients with diabetic nephropathy and provides an additional mechanism by which it may lead to poor outcomes.

## Background

Diabetic nephropathy is the commonest cause of end-stage renal disease (ESRD). Malnutrition in patients with ESRD is associated with increased morbidity and mortality. Nutritional decline begins long before patients with progressive CKD become dialysis dependent [1,2] which in part is related to the spontaneous reduction in dietary protein and caloric intake with reduction in glomerular filtration rate (GFR) [3-8].

Recent studies point out that chronic subclinical inflammation in patients with CKD is associated with an increase in REE [9]. Other studies, done in patients without CKD, show that oxidative stress is not associated with increase in REE [10]. Smoking is associated with both an increase in oxidative stress and subclinical inflammation, thus serves as a model to study the effect of oxidative-inflammatory state on REE [11]. Smoking may therefore compound the oxidative stress and the inflammatory state in patients with CKD [2,12-14]. Whether smoking-induced oxidative-inflammatory state is sufficient to account for elevated REE is not known.

Although smoking-induced increase REE is thought to be acute and transient – in part due to nicotine-induced sympathoadrenal activation – more recent studies point to a chronic elevation in REE [15]. In epidemiological studies, increased REE is seen in young women who smoke compared to women who do not, even after controlling for differences in body size and overnight abstinence from smoking [16]. Smoking cessation often leads to weight gain [17]. Thus, smoking may accelerate malnutrition via accelerating attrition in renal function in addition to elevating REE.

In this study we asked the question whether smokers with diabetic nephropathy have an increased REE compared to non-smokers with equally severe diabetic nephropathy. If so, is the increased REE mediated by the pro-oxidant and inflammatory state.

## Methods

### Subjects

Thirty-five clinically stable Veterans with diabetic nephropathy were recruited from the Renal Clinic of the Roudebush VA Medical Center, Indianapolis, IN after review and approval of protocol by the Institutional Review Board of Indiana University and VA Research and Development Committee. Signed written informed consent was obtained from each participant.

Patients with type 2 diabetes requiring treatment with oral hypoglycemic drugs or insulin, were required to have a urine protein/creatinine ratio of >1.0 g/g on a single voided specimen and a creatinine clearance of >20 mL/min by Cockcroft-Gault formula. All patients were participating in a randomized clinical trial examining the influence of pioglitazone compared to glipizide on reduction of proteinuria. Exclusion criteria included the presence of liver disease, NYHA Class III or IV heart failure, unstable angina, myocardial infarction or stroke in the previous 3 months, NSAID use, or body mass index of ≥40 kg/m^2^.

Patients underwent a standardized history and physical examination that included assessment of medications and control of diabetes mellitus by hemoglobin A_1_C. Current smoking status was ascertained by a yes or no response. The following measurements were made in 32 of 35 subjects on two occasions four months apart; in the remaining three only one measurement was made:

### Glomerular filtration rate

GFR was measured by iothalamate clearance using a continuous subcutaneous infusion at a rate of 125 μL/hour that was started after a bolus intravenous injection 24 hour prior to actual measurements [18]. The following day, six samples of plasma were collected at times 0, 1.0, 1.5, 2.0, 2.5 and 3.0 hours while the subjects were water loaded. Plasma iothalamate concentrations were analyzed using a previously reported high-performance liquid chromatography technique [19]. The ratio of infusion rate to steady-state plasma concentration yielded the iothalamate clearance. The average of these six collections was expressed as the GFR.

### Resting energy expenditure

Seated measurements were made in the morning after an overnight fast. Subjects were asked not to drink or eat anything and refrain from smoking after midnight. They were asked only take half the usual dose of insulin if they were on insulin. Two indirect calorimetric measurements were made 4 months apart in 32 patients and one measurement in the remaining 3 patients.

REE was calculated using open circuit indirect calorimetry in a computerized metabolic system; the gas concentrations of O_2 _and CO_2 _were measured in real time using as gas mass-spectrophotometer (Marquette Electronics, Inc, Milwaukee, WI) connected to a computer. The flow rate of the inhaled and exhaled air was simultaneously measured using a flow meter, interfaced to an analog to digital conversion board with data sampled at 20 Hz and stored to a memory file. The data was analyzed using First Breath Software (First Breath Inc., Ontario, Canada) to calculate oxygen consumption and carbon dioxide production in mL/min. The modified Weir's equation (REE (Kcal/day) = [VO_2 _mL/min (3.94) + VCO_2 _mL/min (1.11)] × 1.44) [20] was used to calculate the REE. Data were collected for 30 minutes and for the purposes of analysis the first 5 minutes of the collection were discarded since they did not represent steady state.

### Lean body mass

To relate REE to differences in body composition between smokers and non-smokers, lean body mass was measured by anthropometric and body impedance spectroscopy.

#### Anthropometric method

Skinfold thickness measurements were taken in triplicate for biceps, triceps, subscapular and supra-iliac areas by a single observer using Lange skin-fold calipers (Beta Technology Inc, Cambridge, MD, USA) and lean body mass (FFM SFT) was calculated by Durnin and Womersley formula [21].

#### Body impedance spectroscopy

Body composition was measured using bioelectric impedance analysis (BIA). A BIA-106 Spectrum II analyzer (RJL Systems, Inc., Clinton Twp. MI) was used and data was collected using Weight Manager Version 2.0 software (RJL Systems, Inc., Clinton Twp. MI). The technique of BIA involved introducing an imperceptible 800 μA alternating current at 50 kHz through signal introduction cables placed with pre-gelled electrodes on the ulnar head and medial malleolus. Sensing electrodes placed at the ipsilateral 3^rd ^metacarpal and 2^nd ^metatarsal, determined the electrical resistance and reactance. Lean body mass was calculated from estimates of total body potassium.

### Protein nitrogen appearance

Two 24-hour urine samples were collected for the measurement of creatinine, urea, sodium, potassium, chloride and protein. The average of the two 24-hour excretion rates was used for analyses. Dietary protein intake was estimated from the urea nitrogen appearance rate (UNA), calculated as follows [22]: Protein intake (g/day) = 6.25[urine urea nitrogen (UUN) (g/day) + 0.031 × body weight(kg)]

### Oxidative stress markers

#### Malondialdehyde (MDA)

Malondialdehyde (MDA), a lipid hydroperoxide, is formed by β-scission of peroxidized polyunsaturated fatty acids and was measured by high performance liquid chromatography following derivatization with thiobarbituric acid (TBA) as reported previously [23]. Urinary MDA was analyzed in three spontaneously voided specimens over a period of 3 hours. Each specimen was analyzed for MDA and creatinine. The MDA/creatinine ratio was averaged and used further for statistical analysis. Blood was collected in tubes containing EDTA at first and last visits. Plasma was separated by centrifugation and stored at -84°C until analysis

#### Carbonyl measurements

Oxidation of plasma and urine proteins were measured by analysis of Western blots with slight modifications of a previous report [24]. Total protein was determined using bicinchoninic acid (BCA-1 Protein Assay Kit, Sigma, St. Louis). Urine protein was measured by the dye binding method using a complex of pyrogallol red and molybdenum acid (QuanTtest Red Total Protein Assay System, Quantimetrix, Redondo Beach, CA). Plasma was diluted 1:25 (v:v) with phosphate-buffered saline (PBS), one aliquot of the diluted sample was derivatized and another prepared as an underivatized control using the OxyBlot protein oxidation detection kit (Intergen, Purchase, NY, USA) Urine samples were derivatized with dinitro phenyl hydrazine (DNP) or control reagent similarly except that samples were not diluted prior. Derivatized and underivatized plasma or urine samples were loaded on electrophoresis gels in volumes calculated to give 5 μg protein per sample and electrophoresed according to the method of Laemmli on 4 to 20%gradient SDS-PAGEgels (Bio-Rad, Hercules, CA, USA) for 60 minutes at 200 V. Following electroblotting to 0.2 μ nitrocellulose for 60 volt hours, the membrane was blocked with subsequent immunoblotting using OxyBlot Kit methods and reagents. Bands were visualized with chemiluminescence and captured on film and analyzed densitometrically using dedicated software using a GelLogic 100 System (Kodak, Rochester, NY).

Samples for individual patients before and after therapy (glipizide or pioglitazone), including derivatized and underivatized control, were analyzed on a single Western blot to ensure that responses to therapy were compared under the same analytical conditions. The blot was stained for protein with amido black and the ratio of the density of the carbonyl band vs the corresponding protein band was used to calculate the percent carbonylation of total protein and comparisons before and after therapy made. The carbonyl band corresponding to the molecular weight of albumin was also compared to the corresponding amido black band to generate a ratio to assess the proportion of albumin oxidized.

### Inflammation markers

#### Total leukocyte count (WBC count)

Blood was collected in EDTA containing vacutainers (Beckton-Dickinson) and total leukocyte count calculated by the Coulter method (Coulter STK-S, Coulter Electronics, Inc., Hialeah, FL) by our clinical laboratory.

#### C-reactive Protein (CRP)

CRP was measured by Cobas Integra 400 autoanalyzer using a particle enhanced turubidimetric assay (Cobas Integra C-Reactive Protein Latex, Roche Diagnostics, Indianapolis, IN). The intra-assay coefficient of variation was 1.8% and the inter-assay coefficient of variation was 2.9% at a mean level of 0.62 mg/dL CRP.

#### Interleukin 6 (IL-6)

Interleukin 6 (IL-6) was assayed in plasma using a sandwich ELISA (Quantikine^® ^kit for Human IL-6 Immunoassay; R&D Systems, Minneapolis, MN). A standard curve was generated using a linear curve-fit. The correlation coefficient for standards was greater than 0.99 and the lowest detectable limit 0.039 pg/ml in undiluted plasma. The intra-assay coefficient of variation was 7.8% and the inter-assay coefficient of variation was 7.2%.

#### Tumor Necrosis Factor-α (TNF-α)

Tumor Necrosis Factor-α (TNF-α) was assayed in plasma using a sandwich ELISA (Quantikine^® ^kit for Human TNF-α Immunoassay; R&D Systems, Minneapolis, MN). A standard curve was generated using a linear curve-fit. The correlation coefficient for standards was greater than 0.99 and the lowest detectable limit 0.12 pg/ml in undiluted plasma. The intra-assay coefficient of variation was 5.9% and the inter-assay coefficient of variation was 12.6%.

#### Urinary Monocyte Chemotactic Protein-1 (MCP-1)

MCP1 was assayed in urine using a sandwich ELISA (Quantikine^® ^kit for Human MCP1 Immunoassay; R&D Systems, Minneapolis, MN). Corrections were made for concentration and values expressed as pg MCP1 per mg creatinine. A standard curve was generated using a four parameter logistic curve-fit. The correlation coefficient for standards was greater than 0.99 and the lowest detectable limit 0.7 pg/ml in 1:2 diluted urine. The intra-assay coefficient of variation was 2.5 ± 3.0% and the inter-assay coefficient of variation was 5.6 ± 4.2%.

### Statistical analysis

Differences in baseline characteristics were compared by a Chi-squared or unpaired *t*-test. To determine the relationship between smoking status and REE a mixed model ANOVA was used to account for correlated observations within subject. The dependent variable was REE/kg body weight; smoking status was used as the fixed effect variable and the subject number as the random effect variable. Clinical, oxidative stress and inflammation markers were used as covariates as noted. The adjusted least square means and 95% confidence intervals obtained form this ANOVA model are reported. SPSS for Windows (version 13, SPSS, Chicago, IL) was used for all analyses. Two sided p-value of less than 0.05 was taken as significant.

## Results

The clinical and laboratory characteristics of the study population are presented in Table 1. In keeping with a diagnosis of diabetic nephropathy, the population was obese, required multiple drugs for treatment of hypertension and had a high prevalence of vascular disease. Smokers and non-smokers were well matched, except in that smokers were younger and had higher baseline glomerular filtration rate.

Body composition, as measured by body impedance spectroscopy and skin-fold thickness assessment, was no different between smokers and non-smokers (Table 2).

Table 3 shows markers of oxidative stress, inflammation, and other metabolic parameters. Plasma and urinary MDA were significantly increased in smokers reflecting the heightened state of oxidative stress. However, protein oxidation markers were similar in the two groups. C-reactive protein was also significantly increased in smokers, reflecting the inflammatory state. Plasma interleukin-6 was marginally higher in smokers, however tumor necrosis factor-α did not reach statistical significance. Urine MCP-1 excretion was nearly equal in smokers and non-smokers. Thyroid stimulating hormone reflecting the overall metabolic state was also no different between the smokers and non-smokers. GFR was significantly higher in smokers at 46.7 mL/min/1.73 m^2 ^compared to non-smokers at 29.1 mL/min/1.73 m^2^.

**Figure 1:**
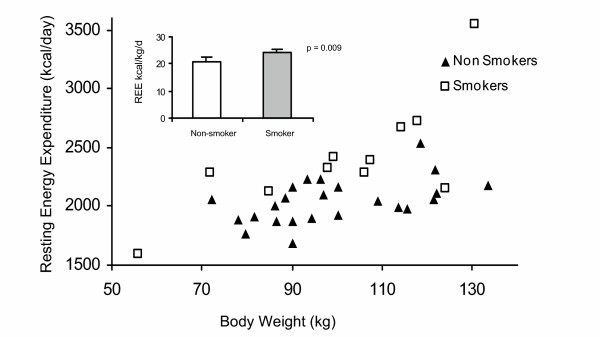
Resting energy expenditure is plotted against body weight. A linear relationship is seen for smokers and non-smokers. Inset shows that the mean unadjusted REE in smokers is higher by 3.26 kcal/kg/day (p = 0.009)

Body weight is an important determinant of REE in smokers and non-smokers (Figure 1). For any given weight, smokers had a higher REE compared to non-smokers. Unadjusted increase in REE was 3.26 kcal/kg/d in smokers compared to non-smokers (p = 0.009). When adjusted for baseline differences in age, GFR, the oxidative stress markers MDA in urine and plasma, and C-reactive protein did not diminish the elevated REE attributable to smoking (Table 4).

There was no independent relationship between MDA/creatinine ratio and REE (partial correlation coefficient = 0.163 (p = 0.19)) or between CRP and REE (partial correlation coefficient = 0.177, (p = 0.16)) after accounting for correlated measurements. None of the other markers of inflammation or oxidative stress achieved statistical significance.

## Discussion

The major finding of this study is that patients with diabetic nephropathy who smoke have an elevated REE after accounting for body weight – the strongest single predictor of REE [25-27]. Smokers had a heightened state of oxidative stress and inflammation. However, neither biomarkers of oxidative stress, nor inflammation had an independent relationship with REE. Furthermore, the association of smoking and elevated REE was not removed when markers of oxidative stress and inflammation were accounted for, strengthening the possibility that oxidative stress and inflammation markers by themselves are insufficient to account for elevated REE in smokers with diabetic nephropathy.

That oxidative stress by itself has little role in elevating REE has been demonstrated in the elderly without kidney disease [10] Our study supports these observations and extends this to patients with CKD.

The relationships between inflammation, creatinine clearance, and REE have been analyzed by Avesani et al in a cross-sectional study of in 91 non-dialyzed patients with CKD [9]. Consideration of kidney function is important for evaluating REE because the oxygen consumption of the kidneys is high – estimated to be between 5.6% and 20% of the total metabolic rate [28,29] – and failing kidneys may have reduced oxygen consumption contributing to reduced REE [29,30]. REE may also decrease as an adaptation to the decreased energy intake or accumulation of uremic toxins that depresses metabolic activity. However, studies are not conclusive regarding this observation. Whereas some studies have shown decreased [31,32] REE others have shown no change [33] or even increased [34] REE in CKD. Avesani et al did not find any relationship between quartiles of creatinine clearance and REE but they were amongst the first to propose inflammation as a cause of elevated REE in CKD [35]. Those in the highest tertile of CRP – and accordingly most inflamed – had an unadjusted REE that was higher compared to the lower tertiles of CRP. Consideration of smoking status as a cause of raised REE has not been reported in any of the above studies. It is possible that the association of increased CRP with REE may simply be due to overrepresentation by smokers in the highest tertile of CRP. Our study suggests consideration of smoking as a covariate in analyses of REE in patients with CKD.

Given that GFR can have a variable influence on REE, as noted above, it is important to account for the effect of GFR in studies dealing with REE in patients with CKD. Smokers in this study had a statistically increased GFR compared to non-smokers which is similar to what has been reported for a large population based study from France [36]. However, adjustment for GFR did not remove the effect of smoking to elevate REE. Other adjustments, such as that for age, CRP, urine and plasma MDA also did not remove the association of increased REE with smoking. Thus, other unmeasured factors may account for the elevated REE in smokers.

The effect of cigarette smoking on REE are thought to be acute and transient and related to sympathetic activation [15]. It should be noted that this study did not examine the acute effect of smoking, rather the chronic effect of smoking. The large effect of smoking on REE was somewhat unexpected. In one epidemiologic study, women who smoked had a statistically significant increase in REE [16]. The magnitude of the effect of smoking was 68 kcal/d which was approximately one-third of the effect size reported in the present study [16]. In another study, black women who smoked were found to have a greater baseline REE compared to white women [37]. Specifically, amongst black women, heavy smoking was associated with 218 kcal/d higher REE and moderate smoking with 177 kcal/d higher REE compared to non-smokers. Although studies on the effect of smoking in diabetic nephropathy or those with CKD have not been reported, the results of this study are in line with those reported by others in populations without CKD [16,37] and extend these observations to patients with CKD. Exactly how chronic smoking leads to increased REE is not clear from present investigations [38].

There are some limitations of this study. First, the sample size was relatively small and limited to men. Nevertheless, repeated observations in the same patient improved the precision of the measurements and demonstrated a highly significant difference between smokers and non-smokers. Second, diabetes control was less than optimal in some patients that can elevate resting energy expenditure by itself. However, adjustment for diabetes control did not mitigate the significance of the results. Third, we did not quantify the amount and duration of smoking that may have an independent effect on REE [37]. Finally, these data may not be applicable to women.

In summary, this study demonstrates higher REE in smokers with diabetic nephropathy after adjusting for body weight. These findings generate the hypothesis that smokers with diabetic nephropathy would be most prone to protein-energy malnutrition at the onset of ESRD and add to other health-risks of smoking in this population[31,33,34,39]. Aside from increasing REE, smoking is known to suppress appetite, which given the anorexic state of uremia, can produce malnutrition by two ways – lowering intake and increasing energy expenditure. Smoking should be considered as a cause of increased REE in patients with CKD when analyzing the effect of other variables such as inflammation and GFR. Longitudinal studies are required to better define the complex relationship between REE, falling renal function, oxidative stress and inflammation in patients with CKD.

## Competing interests

The author(s) declare that they have no competing interests.

## Authors' contributions

The author designed and secured funding for the study, trained the study personnel in the collection of the data, recruited the patients and finally analyzed the data and wrote the manuscript.

## Pre-publication history

The pre-publication history for this paper can be accessed here:



## Figures and Tables

**Table 1:** Clinical and Laboratory Characteristics of Patients with Type 2 Diabetic Nephropathy

Parameter	Non-smokers Mean ± SD or N (%)	Smokers Mean ± SD or N (%)
N	24	11
Age (y)	69.1 ± 7.0	61.5 ± 9.3 *
Males/Females	24/0	11/0
Race White/Black	19/5	9/2
Weight (kg)	97.8 ± 16.8	102.5 ± 23.4
BMI (kg/m^2^)	32.6 ± 4.9	32.6 ± 6.6
Serum albumin (g/dL)	3.8 ± 0.33	3.63 ± 0.30
Serum Creatinine (mg/dL)	2.75 ± 1.21	1.80 ± 0.74 *
BUN (mg/dL)	46.5 ± 25.6	38.3 ± 21.8
Hemoglobin (g/dL)	13.3 ± 1.6	13.5 ± 2.4
24 hour urine protein (g/d)	3393 ± 2522	4423 ± 4385
Iothalamate clearance (GFR) (mL/min/1.73 m^2^)	28.9 ± 13.8	47.2 ± 34.8 *
Duration of diabetes (yr)	15.7 ± 9.1	13.3 ± 9.0
Insulin use	15 (63%)	6 (55%)
Hemoglobin A_1_C (%)	7.57 ± 2.02	8.98 ± 2.93
Waist/Hip Ratio	1.00 ± 0.07	1.04 ± 0.07
Antihypertensive medications	4.3 ± 1.6	3.9 ± 1.9
ACE inhibitor or Angiotensin receptor blocker use	21 (88%)	8 (73%)
Total Cholesterol (mg/dL)	184 ± 51	223 ± 87
LDL Cholesterol (mg/dL)	100 ± 44	116 ± 24
HDL Cholesterol (mg/dL)	42 ± 11.1	46 ± 11.4
Vascular Disease (coronary, cerebrovascular or peripheral vascular disease)	17 (71%)	7 (64%)

* p < 0.05

**Table 2:** Body Composition Analysis by Body Impedance Spectroscopy and Skin Fold Thickness

**Parameter**	**Non-Smokers N = 24**	**Smokers N = 11**
**Body Weight (kg)**	99.2 (91.5 – 106.9)	101 (89.6 – 112.4)
**Phase angle**	5.70 (4.46 – 6.93)	5.12 (3.30 – 6.94)
**Vector length**	227 (210 – 244)	202 (177 – 227)
**Fat free mass (kg)**	74.4 (68.2 – 80.6)	81.5 (72.4 – 90.6)
**Total body water (L)**	55.3 (50.9 – 59.8)	60.6 (54.1 – 67.1)
**Fat mass (kg)**	25.4 (20.1 – 30.7)	21.3 (13.5 – 29.0)
**Total body potassium (mEq)**	3981 (3650 – 4313)	4130 (3641 – 4618)
**Body cell mass (kg)**	33.2 (30.4 – 35.9)	34.4 (30.3 – 38.5)
**Extracellular mass (kg)**	41.3 (36.4 – 46.1)	46.9 (39.8 – 54.1)
**Intracellular water (L)**	30.3 (27.7 – 32.8)	31.4 (27.7 – 35.1)
**Extracellular water (L)**	25.1 (21.6 – 28.5)	29.0 (23.9 – 34.1)
**Fat free mass from Skin fold thickness (kg)**	53.4 (50.2 – 56.6)	56.4 (51.6 – 61.2)

Least square mean values with 95% confidence intervals are calculated from the mixed model ANOVA model as described in methods. None of the comparisons were significantly different between the groups.

**Table 3:** Comparison of REE, Inflammation and Oxidative Stress Markers in Smokers and Non-smokers

Parameter	**Non-smokers**	**Smokers**	**Difference (Smokers – non-smokers)**	**p**
REE/kg body wt.	21.0 (19.7 – 22.3)	24.3 (22.3 – 26.2)	3.26 (0.87 – 5.6)	0.009
Respiratory Quotient	0.85 (0.83 – 0.87)	0.81 (0.78 – 0.85)		NS
Oxidative Stress Makers				
Plasma malondialdehyde (μM/L)	0.94 (0.79 – 1.08)	1.20 (0.98 – 1.41)	0.26 (0.0 – 0.52)	0.05
Urine malondialdehyde/urine creatinine ratio (μM/g creatinine)	3.36 (2.44 – 4.28)	6.33 (4.95 – 7.72)	2.97 (1.31 – 4.64)	0.001
Plasma total carbonyl (DU)	0.818 (0.728 – 0.908)	0.791 (0.658 – 0.924)		NS
Urine total carbonyl (DU)	0.454 (0.336 – 0.571)	0.444 (0.270 – 0.617)		NS
Plasma albumin carbonyl (DU)	1.425 (1.278 – 1.572)	1.441 (1.225 – 1.658)		NS
Urine albumin carbonyl (DU)	0.598 (0.438 – 0.758)	0.591 (0.356 – 0.827)		NS
Inflammation Markers				
White blood cell count (number/μL)	7897 (6885 – 8908)	9139 (7647 – 10,631)		NS
C-reactive protein (mg/dL)	0.80 (0.37 – 1.23)	1.77 (1.13 – 2.40)	0.97 (0.21 – 1.74)	0.014
Interleukin-6 (pg/mL)	2.13 (1.48 – 2.78)	3.28 (2.31 – 4.25)	1.15 (-0.02 – 2.32)	0.053
Tumor necrosis factor-α (pg/mL)	4.05 (3.05 – 5.05)	3.89 (2.40 – 5.38)		NS
MCP-1/Creatinine (pg/mg)	398 (288 – 508)	399 (228 – 569)		NS
Other variables				
HbA1C (%)	7.46 (6.64 – 8.27)	8.73 (7.52 – 9.94)		NS
Plasma glucose (mg/dL)	132 (110 – 153)	168 (136 – 200)		NS
Protein nitrogen appearance rate (g/kg/d)	0.76 (0.68 – 0.83)	0.81 (0.71 – 0.92)		NS
Iothalamate clearance (mL/min/1.73 m^2^)	29.1 (19.6 – 38.6)	46.7 (32.7 – 60.6)	17.6 (0.7 – 34.6)	0.042
24 hour urine protein (mg/d)	3384 (2180 – 4588)	4189 (2410 – 5967)		NS
Thyroid stimulating hormone	2.32 (1.87 – 2.78)	2.04 (1.37 – 2.71)		NS

Values reported are least square mean values with 95% confidence intervals are calculated from the mixed model ANOVA model as described in methods. DU stands for densitometric units, REE for resting energy expenditure, MCP-1 for monocyte chemotactic protein-1.

**Table 4:** Increase in resting energy expenditure in unadjusted and adjusted models associated with smoking

**Adjusted variable**	**Resting Energy Expenditure (kcal/kg/d)**	**95% confidence interval of the estimate**	**P value**
**None (Unadjusted model)**	3.26	0.87 – 5.6	0.009
**Age**	3.70	1.05 – 6.35	0.008
**Age + GFR**	3.67	0.94 – 6.40	0.01
**Age + GFR + Urine MDA/Cr**	3.95	1.06 – 6.84	0.009
**Age + GFR + Urine MDA/Cr + CRP**	3.71	0.74 – 6.69	0.016
**Age + GFR + Urine MDA/Cr + CRP + Plasma MDA**	3.48	0.57 – 6.39	0.021
**Age + GFR + Urine MDA/Cr + CRP + Plasma MDA + HbA_1_C**	3.51	0.59 – 6.45	0.020

Adjustment for covariates are evaluated at the means which were age = 66.7 years, GFR = 35.6 mL/min/1.73 m^2^, Urine MDA/Cr = 4.3 μmol/g, Plasma MDA 1.03 μmol/L, CRP = 1.00 mg/dL, HbA_1_C = 7.78%.
